# The roles and influence of actors in the uptake of evidence: the case of malaria treatment policy change in Uganda

**DOI:** 10.1186/s13012-014-0150-8

**Published:** 2014-10-08

**Authors:** Juliet Nabyonga-Orem, Miriam Nanyunja, Bruno Marchal, Bart Criel, Freddie Ssengooba

**Affiliations:** WHO Regional Office for Africa, Brazzaville, Congo; WHO Uganda Country Office, Kampala, Uganda; Institute of Tropical Medicine Antwerp-Belgium, Nationalestraat 155, Antwerp, 2000 Belgium; School of Public Health, Makerere University, Kampala, Uganda

**Keywords:** Knowledge translation, Stakeholders, Malaria, Treatment policy change

## Abstract

**Background:**

Uganda changed its malaria treatment policy in response to evidence of resistance to commonly used antimalarials. The use of evidence in policy development—also referred to as knowledge translation (KT)—is crucial, especially in resource-limited settings. However, KT processes occur amidst a complex web of stakeholder interactions. Stakeholder involvement in evidence generation and in KT activities is essential. In the present study, we explored how stakeholders impacted the uptake of evidence in the malaria treatment policy change in Uganda.

**Methods:**

We employed a qualitative case study methodology involving interviews with key informants and review of documents. A timeline of events was developed, which guided the purposive sampling of respondents and identification of relevant documents. Data were analysed using inductive content analysis techniques.

**Results:**

Stakeholders played multiple roles in evidence uptake in the malaria treatment policy change. Donors, the Ministry of Health (MoH), service providers, and researchers engaged in the role of evidence generation. The MoH, parliamentarians, and opinion leaders at the national and local levels engaged in dissemination of evidence. The donors, MoH, researchers, and service providers engaged in the uptake of evidence in policy development and implementation. Stakeholders exerted varying levels of support and influence for different reasons. It is noteworthy that all of the influential stakeholders were divided regarding the best antimalarial alternative to adopt.

**Conclusion:**

Our results showed a diverse group of stakeholders who played multiple roles, with varying levels of support and influence on the uptake of evidence in the malaria treatment policy change. For a given KT processes, mapping the relevant stakeholders and devising mechanism for their engagement and for how to resolve conflicts of interest and disagreements *a priori* will enhance uptake of evidence in policy development.

**Electronic supplementary material:**

The online version of this article (doi:10.1186/s13012-014-0150-8) contains supplementary material, which is available to authorized users.

## Background

Since 2001, Uganda has changed its malaria treatment policy twice, after efficacy studies demonstrated significant resistance against first-line antimalarials [1],[2] beyond the thresholds at which the World Health Organisation (WHO) recommends policy change [3]. The use of evidence in policy development is of critical importance, especially in resource-limited settings; however, data suggest that its potential has yet to be fully realised [4],[5]. Here, evidence is broadly defined to include research study results (both published and unpublished), findings of monitoring and evaluation (M&E) studies and population-based surveys, Ministry of Health (MoH) reports, community complaints, and clinician observations [6],[7]. The application of such evidence in policy development is also referred to as knowledge translation (KT), which the Canadian Institute of Health Research defines as “a dynamic and iterative process that includes synthesis, dissemination, exchange and ethically sound application of knowledge to improve health, provide more effective health services and products, and strengthen the health care system” [8]. Efforts to improve KT—including developing KT models and implementing several KT activities—have produced mixed results [9]-[12].

KT processes occur amidst a complex web of interactions between stakeholders, who are hereby defined as individuals who, or institutions which are affected by the policy change, directly influence it, or have an interest in the outcome even when not directly involved [13]. In this article, we use the words stakeholders and actors interchangeably. Scholars have noted that the roles played by stakeholders and their level of influence, support, and interactions have an important impact on how evidence influences policy [14],[15]. Mori et al. documented instances where essential medicines were selected based on the experience and discretionary judgment of experts, despite the availability of hard efficacy data [4]. Cases where stakeholder involvement has delayed the translation of evidence into policy have also been documented [16],[17]. For example, delays have been attributed to researchers devoting more time to generating evidence than to disseminating their results [17].

The available literature highlights several potential roles that stakeholders may play in KT. For example, civil society organisations (CSOs)—which here are defined as formally organised non-profit groups concerned with public interests [18]—reportedly advocate for evidence uptake, undertaking research, mobilising communities to demand evidence implementation, and implementing evidence in their own programmes [18]-[20]. On the other hand, communities can participate in research processes as respondents but also in setting the research agenda [21],[22]. The media can be an effective ally in evidence dissemination, community mobilisation, and shaping public opinion [21],[23],[24], while policymakers are responsible for translating evidence into policies and putting the necessary KT platforms into place for engagement among stakeholders [9],[24]. Donors fulfil the main role of funding the research, KT activities, and implementation of research findings [9],[25]. Finally, researchers can intervene as stakeholders who generate evidence [7],[11],[25].

Tomlinson et al. point out that the specific composition, roles, and impact of stakeholders in KT are influenced by the context within which KT processes take place and the nature of the policy [26]. For example, Woelk et al. studied the uptake of evidence on malaria control and treatment of eclampsia in three Southern African countries and documented a wide range of international stakeholders influencing the former, while mainly international academic networks influenced the latter [27]. Indeed, actors have played different KT roles with regard to malaria treatment policy changes. For example, researchers and policymakers were instrumental in synthesising and disseminating evidence on the effectiveness of artemisinin combination therapies (ACTs) in Burkina Faso [28], while policymakers have been weak in evidence synthesis in other KT processes [24]. In Sudan, an NGO took the lead in putting a KT platform into place [29], which has been the role of ministries of health in other instances [9]. In Tanzania, pharmaceutical manufacturers and medicine traders reportedly influenced malaria treatment policy changes through their opposition to the change from chloroquine (CQ) to sulfadoxine/pyrimethamine (SP), as the former had invested in continued CQ production, while the latter still had large CQ stocks [16]. The WHO has been instrumental in providing technical guidance on malaria treatment policies at both the global and national levels [23],[29], which is not the case in other policy processes—for example, health financing.

The present study is part of a larger study that seeks to enhance our understanding of how we can improve evidence uptake in health policy development. In our previous work, we explored the roles, relationships, and interactions of key stakeholders involved in KT in Uganda, without specific reference to a piece of evidence or a policy. Nabyonga Orem et al. showed that stakeholders in KT were perceived to play both positive and negative roles, as well as identified the challenges that they had to overcome to effectively play the positive roles (Table 1) [30].

**Table 1:** **Summary of roles of stakeholders in KT related to health policy development in Uganda**

Stakeholder	Roles	Challenges to overcome	Links that need to be built
CSOs	-Uptake of evidence in policy development and implementation	-Need skills in navigating the political terrain, networking, and engaging policymakers	-Links among CSOs, researchers, and policymakers
	-Dissemination of evidence	-Must be provided with clear and simplified formats to avoid misrepresenting the evidence	
	-Advocating for evidence implementation	-Must be funded independently of the government	
Policymakers	-Uptake of evidence in policy development	-Capacity to synthesise evidence	-Links between policymakers and researchers
	-Establishing platforms for KT and playing a leadership role		
Media	-Dissemination of evidence	-Need to be provided with evidence in simplified and preferably written formats	-Links between researchers and the media
Parliamentarians	-Dissemination of evidence	-Require targeted dissemination to parliamentarians	-Links among researchers and parliamentarians
	-Community mobilisation		
Communities	-Participation in research processes	-Putting into place community structures to enable their participation in research processes	-Links between communities and researchers
Donors	-Funding research and implementation of evidence	-Governments must establish structures for developing research agendas through inclusive participatory partnerships	
Researchers	-Evidence generation	-Focusing on academic interests	

Source: Nabyonga Orem et al. [30].

In the present article, we explore how different stakeholders have shaped evidence uptake in relation to malaria treatment policy change in Uganda, with specific assessment of the roles they played and their level of influence and support. Furthermore, we investigated the extent to which the previously identified roles of stakeholders in KT in Uganda [30] differed from their roles specifically in relation to malaria treatment policy change. For this project, we employed a case study approach, using qualitative methods involving interviews with key informants (KIs) and review of documents. The case analysed was the malaria treatment policy change from CQ/SP to ACT—more specifically, artemether-lumefantrine (AL)—which transpired over a period of 25 months between March 2004 and April 2006 in Uganda.

## Methods

This case study employed qualitative methods to explore how the involved stakeholders impacted evidence uptake in the malaria treatment policy change process. The case study approach was chosen based on the need to understand complex contextual issues [31]. Data were collected between June 2012 and August 2013. To enhance the validity of our results, we employed multiple data collection methods and member checking [31]. Prior to finalisation, preliminary results were reviewed by stakeholders who were central to the policy case: two from the WHO and two from the MoH. Recall bias was ameliorated by interviewing a wide range of knowledgeable stakeholders and by the use of multiple data sources [31].

**Figure 1:**
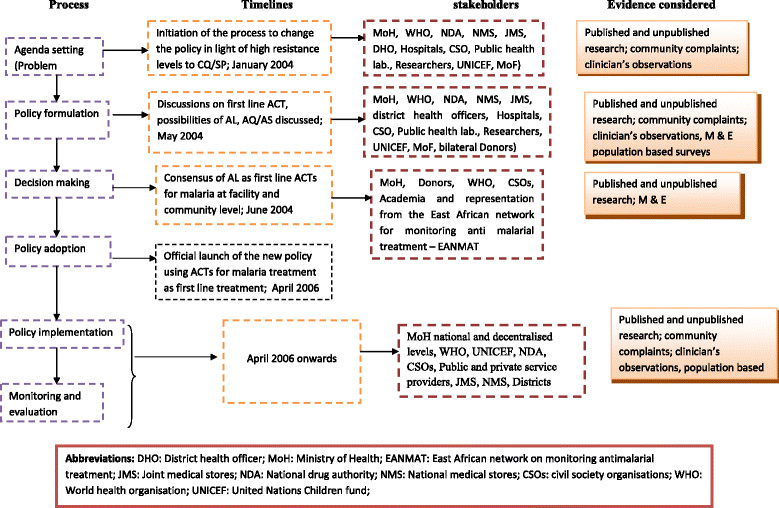
**Process, timelines, and stakeholders involved in the malaria treatment policy change.**

A timeline of key events was drawn based on the review of documents, in consultation with two persons from the WHO and two persons from the MoH who had each held malaria-focused positions for over 10 years. This timeline guided the identification of key milestones, the involved processes, the key documents to be reviewed, and the institutions involved, which subsequently informed the selection of respondents (Figure 1).

### Stakeholder analysis

A stakeholder analysis was undertaken to assess the roles, level of support, and influence of the actors regarding evidence uptake in the malaria treatment policy change. Stakeholder analysis is a powerful tool that can be retrospectively used to understand the roles, interests, and influences of the different stakeholders in the evolution of policy context and processes [32]. Here, our research team drew upon the work of Eden and Ackermann [33] and Bryson [34] in undertaking the analysis and classifying the stakeholders using the influence/power-support/interest grid.

### Selection of respondents

Using the timeline of key events, we identified institutions that were involved in the policy process. We selected KIs using purposive sampling with the main criterion being their involvement in either research, design, or implementation of the malaria treatment policy change [35]. From each of the key institutions, we selected the focal persons involved in the policy change process and employed the snowballing technique to identify other key respondents until reaching descriptive saturation [36],[37]. Some of the identified respondents had since moved on to other employment or retired, and these persons were categorised under the institutions that they worked for at the time of the policy change. The identified focal researchers were selected for interviews if they had been involved in malaria research and had provided evidence that was considered in the policy change process. Emphasis was placed on collecting their perceptions in line with the study questions, beyond what they may have published in scientific papers and research reports.

To obtain perceptions from across the spectrum of the health-care delivery system, we purposively selected two districts of high malaria endemicity [38], based on proximity and presence of a regional referral hospital (Jinja district) or general hospital (Mpigi). Within these districts, two hospitals and two lower level facilities (one public and one private not-for-profit in both districts) were purposively selected based on proximity and our desire to include different levels of the health-care system. At the district level, we purposively selected the district health officer and a member of the district health team in charge of supervising health facilities within the district. Finally, we purposively selected the medical superintendent or health centre employee in-charge and one clinical staff member responsible for the outpatient department at each health facility, as these employees interface with patients on a daily basis and are more likely to know the malaria burden, community health-seeking behaviours, and interfaced often with the supervising teams.

The selected respondents included public policymakers, donor representatives, media, CSOs, researchers, and representatives of the pharmaceutical sector. We also interviewed managers of health services at the district level, health-care providers from the public and private not-for-profit health facilities, the National Medical Stores (NMS) in charge of medicine procurement and distribution and the National Drug Authority (NDA) in charge of medicine regulation (Table 2).

**Table 2:** **Key informants**

	Institution	Number of respondents	Average number of years in post
	Donors	3	8
Public sectors	National level MoH	10	11
	National Medical Stores (NMS)	1	3
	National Drug Authority (NDA)	1	6
	Service providers	4	7
	Managers at district level	4	9
	Researchers at universities	1	8
Private sectors	Civil society organisations	3	9
	Researchers from private research institutions	1	7
	Media	1	8
	Private pharmaceutical sector	1	5
	Service providers^a^	3	6
Total number of respondents		31	

^a^One of the selected districts did not have a private not-for-profit hospital.

KIs were interviewed using an in-depth interview guide that comprised open-ended questions designed to elicit the respondents’ perceptions on whether evidence had been used and who the stakeholders were, the roles they played, and their level of interest in and support for evidence uptake in the policy change process. The interview guide was developed by the first author, was reviewed and refined by the research team, and was pretested with two volunteer colleagues in the WHO Uganda office, two technical officers in the MoH, and one researcher from the Makerere University School of Public Health. KIs were contacted by email or telephone and invited to participate in the study. All identified respondents agreed to participate and were interviewed. All interviews were conducted by the first author, in English and face-to-face. The interviews lasted an average of 45 min. During the interviews, the first author made additional notes to record initial findings and impressions that were used to augment the transcribed interviews.

### Selection of relevant documents

The timeline of key events guided the identification of relevant documents to be reviewed. We included a broad range of documents relevant to the case to ascertain the processes involved, the stakeholders, and their roles. All identified documents were retrieved and reviewed. Additional file 1 presents details of the reviewed documents.

### Data analysis

Interviews were recorded, transcribed verbatim, and entered into MS Word software for editing as the first step towards a “formal” analysis. All interviews and reviewed documents were coded using QRS Nvivo Software Version 10. Content analysis techniques were used to construct emerging categories linked to the research issues [39]. In the first step, the first author read all of the transcribed interviews and relevant documents to identify categories of emerging issues with regard to the involved stakeholders, the roles they played, whether they were supportive, and their possible influence on evidence uptake in the policy process. Next, the study team together analysed the transcripts in order to identify categories of emerging issues according to type of stakeholder, which were organised based on research areas. Inductive manifest content analysis was undertaken to assess how respondents perceived and how documents reflected the role(s) played by the different stakeholders, while inductive thematic content analysis was undertaken to assess the level of support and influence of the different stakeholders. Examples are shown in Additional file 2a and b.

Converging issues were again reviewed by the rest of the research team. Where interpretation differed, consensus was achieved through revisiting the raw data and discussions. Where necessary, quotations that best represented emerging issues were slightly edited for flow, while preserving the meaning of the text. The findings from document analysis and from the analysis of KI interviews were integrated throughout the analysis.

Informed consent was obtained from all respondents prior to the interviews. Study participants were informed about the purpose of the study and the scope of issues in the in-depth interview guide. Confidentiality was ensured in data management, and only aggregate information without subject identifiers is reported. All data were secured in a safe location accessible only to the study team. Ethical approval was obtained from the Institutional Review Board of the Institute of Tropical Medicine, Antwerp (Belgium) (IRB number IRB/AC/ac/197) and the Uganda National Council for Science and Technology (number SS 2920).

## Results

We integrated the results from the review of relevant documents and from the interviews with KIs. These findings are presented in three sections, namely, the roles played by the different stakeholders, the stakeholders’ level of support and influence, and other external influences on the uptake of evidence. The respondents reported that evidence informed the malaria treatment policy change, which was supported by the review of documents. For example, documentation of the malaria treatment policy change process in Uganda (MoH 2006) states that “*the decision to change the malaria treatment policy was based mainly on evidence from efficacy studies, which showed high resistance of plasmodium falciparum to CQ*”. Other types of evidence were also used. For example, one research remarked that “*communities complained first `I have been taking this medicine for malaria with no improvement”. This was then picked up by the health workers and then by the scientists*”. Additional different types of evidence were cited in all 18 documents that were reviewed.

### Roles played by the different stakeholders in the uptake of evidence

Throughout the process of policy formulation, decision making, policy adoption, and implementation, stakeholders participated in various task forces charged with the responsibilities of developing the new policy and mainstreaming its implementation in routine processes. The review of documents supported this. Documentation of the malaria treatment policy change process in Uganda (MoH 2006) stated that “*having reached a consensus that the malaria treatment policy needs to change in light of high resistance to the first line anti-malarials, task forces including experts in malaria from the different institutions/agencies were commissioned to work out the policy change and implementation process*”. The available documentation further indicated that the different task forces were expected to use available evidence in their deliberation. For example, the report of the supply chain management task force (MoH 2004) stated that “*the task force calculated the amount of AL required based on the malaria prevalence, health seeking behaviour, and population growth rate*” [40]. Similarly, a report of the task force on treatment guidelines and training approaches (MoH 2004) indicated that their goal was to “*update the current guidelines for treating malaria in line with evidence*”.

The majority of stakeholders played multiple roles in the uptake of evidence in policy development and implementation (Table 3). For example, the donors’ roles encompassed providing funding for evidence generation, participating in research processes through regular updates and discussions of preliminary results, drafting the new policy through their membership in working groups that discussed policy options given the available evidence, and supporting evidence implementation through provision of funds and free medicines.

**Table 3:** **Roles played by the different stakeholders**

	Stakeholders	Roles
Donors	Global Fund	Funding the policy change
	WHO	Providing evidence and technical assistance (TA), participating in the policy process, funding research
	UNICEF	Providing evidence, participating in the policy process
	Presidential Malaria Initiative/USAID	Funding research and participating in the policy process
	Department for International Development (DFID) from the UK	Funding research and participating in the policy process
	China	Providing free medicines (AL)
Pharmaceutical companies	NOVARTIS	Manufacturing the drug
	Private pharmaceutical companies	Importing drugs
Public sector	Top management of MoH	Decision making
	Technical programmes within MoH	Adapting and implementing the policy, participating in research processes, and disseminating evidence
	Ministry of Finance	Providing funding
	Parliamentarians	Disseminating evidence and information on the policy change
	Service providers at national referral institutions	Providing evidence, participating in the policy process
	Service providers at lower levels	Implementing the new policy
	NDA	Regulating medicines
	National Medical Store and Joint Medical Store	Supplying drugs
	Researchers in universities	Providing evidence and participating in the policy process
Community	The community	Beneficiaries of the policy change
Local leaders	Opinion leaders at national level	Disseminating information on the policy change
	Leaders at the local level	Disseminating information on the policy change
Private sectors	CSOs	Participating in policy discussions, research, advocacy, and implementation and monitoring of the new policy
	Private practitioners	Implementing the new policy
	Researchers from private research institutions	Providing evidence and participating in the policy process
	Private sector—companies	Advocacy, publicity, donations

The top management and technical programmes in the MoH reportedly led the policy development process, including discussions on the best options given the available evidence on drug efficacy and health expenditures trends. This was further supported by the review of documents. For example, the concept paper for implementing the ACT policy (MoH 2004) stated that “*the MoH is responsible for overall coordination of the policy change process*”. Technical programmes within the MoH, specifically the national malaria control programme (NMCP), also engaged in research processes. In particular, one researcher remarked that “*At the time of doing the studies, the NMCP was part of this, so they approved the studies. Then at the level of the studies getting approved by the review committees, they were always inquisitive to see whether the NMCP was on board*”. On the other hand, the NDA reportedly played the role of medicine registration and regulation. Again, this was supported by the review of documents, as the report of the workshop on strategies for implementation of the new antimalarial policy (MoH 2006) stated that “*COARTEM was officially registered as an antimalarial medicine by the NDA in June 2006*”.

CSOs played roles spanning from involvement in research by participating in regular updates to incorporating evidence into the new policy via participation in discussions regarding policy options and implementation of evidence through their programmes. Members of parliament were reportedly engaged in dissemination of evidence. Supporting this, the malaria prevention and control handbook for parliamentarians (MoH 2005) stated that “*the development of this handbook is yet another opportunity to provide information to Members of Parliament to enhance their ability to communicate and disseminate information on malaria prevention and control from an informed position*” [41].

Respondents reported that researchers also engaged in policy discussions—particularly when evidence was discussed—to guide selection of the best option, as well as reviewed drafts of the new policy. Opinion leaders at the national and district levels reportedly disseminated information on the policy change, along with reasons necessitating the policy change with reference to evidence on drug resistance, and mobilised communities to take up the new policy. The communication strategy for treatment of uncomplicated malaria using AL (MoH 2004) states that “*Influential persons and leaders at the national and district levels were armed with information on the new treatment and were requested to share information with the community and advocate for compliance*”.

Notably, the role of Novartis—the pharmaceutical company that was manufacturing ACTs—was not reflected in the reviewed documents. It was also striking that communities were not reflected as stakeholders in the reviewed documents, and private practitioners were only involved as trainees in preparation for rolling out the new policy.

### Stakeholders’ levels of support and influence in evidence uptake in the policy process

**Figure 2:**
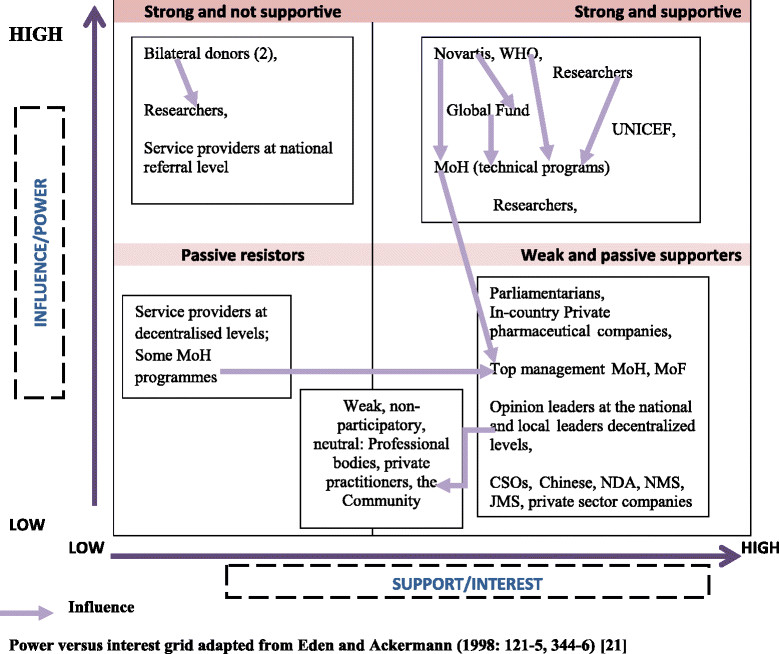
**Support and influence of the different stakeholders involved in the malaria treatment policy change.**

Figure 2 shows the levels of support and influence of the different stakeholders in the evidence uptake in the policy process. Some stakeholders were reportedly very influential because they had significant resources, were highly respected, exercised a strong influence on the MoH, and strongly believed in the evidence, as well as because the decision regarding which options were adopted and the subsequent policy implementation heavily depended on their opinion.

Although the majority of respondents believed that the evidence demonstrated a need to change the malaria treatment policy, there was no consensus regarding the best alternative. Some research groups presented evidence showing that AL was effective, while others had evidence showing the effectiveness of amodiaquine (AQ) and artesunate (AS). A donor respondent reported that “*there was a lot of influence pushing everyone for ACTs but some stakeholders were pushing for amodiaquine (AQ) and artesunate (AS). When AL was selected, the push for AQ and AS continued and the team had to go back and decided to have these as an alternative*”.

Novartis, the pharmaceutical company that manufactures AL, was reportedly supportive of the change to AL. Some respondents reported a possible influence of Novartis on the MoH, on the Global Fund (GF), and in the decision-making process regarding which alternative antimalarial to adopt, as shown in the following quotes:

“*There were some experiences of people being taken to workshops to discuss the malaria treatment policy change and subsequently visiting the factories—the AL manufacturers. This may have influenced the decision to adopt AL.*” MoH respondent

“*The pharmaceutical company Novartis could have been talking to the GF, who were saying that the GF grants should only be used for buying ACTs. This was at a time the MoH was applying for GF round 4. So I think, there could possibly have been some external influence to change to AL.*” Researcher respondent

The MoH was not unified in the process. Some MoH officers were reportedly influential and supportive of the change to AL, while others acted more as passive resistors and were less influential. The NMCP was reportedly very supportive and influential, because the adoption of AL as the first-line antimalarial and its subsequent implementation heavily depended on them. Additionally, the NMCP officers strongly believed in evidence. They closely followed the research results from the sentinel sites and regularly met with the researchers. One researcher remarked that “*at the time that the malaria treatment policy changed from CQ/SP to ACTs, the NMCP was in the hands of two people who both believed in evidence and the researchers worked very closely with malaria control programme leadership.*”

The passive resistors were not convinced of the need to change to ACTs (specifically AL), despite the evidence showing their effectiveness. They thought the decision to change was influenced by donors, as shown in the following quotes:

“*Regarding anti-malaria drugs, we had our own sentinel sites around the country and we were gathering sensitivity data on existing and alternative anti-malarials. So, yes, there was CQ resistance but, we were looking at, I think, AQ as an alternative.*” MoH respondent

“*Although efficacy data showed resistance to CQ, we were not yet ready for a treatment change. We would have moved at the right time, but when the GF insisted that GF grants should only be used to buy ACTs, it became difficult to opt for another alternative.*” MoH respondent

Top management staff of the MoH were noted to be weak and passive supporters. One respondent reported that the top management cadre could have been influenced by the divided opinions at the technical level, stating the following:

“*the technical programmes in the MoH were divided. We had a very strong personality like [X] in the NMCP who was very clear. Then there were people who were doubtful despite availability of evidence. If the technical programmes were very clear maybe even top management would have been convinced and rallied behind the decision. The technical programmes wavered in their positions and that could have had an influence on top management.*” MoH respondent

Researchers were reportedly very influential because they were highly respected even if they were divided. In terms of the research results, there was a consensus regarding the high levels of resistance to CQ/SP; however, there were arguments among researchers as to what alternative drug was most suitable, given the evidence showing effectiveness of both ACT specifically (AL) and, AQ and AS. A donor respondent remarked that “*some researchers did not think we needed to change to ACTs (AL). They were saying this was commercially driven and that other options like AQ and AS would have been more appropriate*”. Some respondents felt that some researchers used evidence to exert pressure, as one journalist made the following remark: “*there was a man called [Y] who wrote an article in the Lancet criticizing the MoH and WHO for sticking with CQ/SP as the first-line treatment. He was saying `look, you are having CQ/SP and both of them were having high resistance rates; so by combining the two you are not making matters any better’*”.

Others questioned the neutrality of the research community, noting that some of the research teams had been funded by donors who did not support choosing AL as an alternative first-line drug. There were suspicions that the researchers were perhaps being influenced, as shown in the following quote:

“*These researchers were funded by donors, and those particular donors had different views on possible alternative anti-malarials. When these researchers presented their results, there was a lot of disbelief initially. People made statements like `Anyway, if you look for the resistance you will find it’. We were using CQ/SP properly and no one was complaining until efficacy studies started reviewing the data. Then we started saying we have resistance to CQ/SP.*” Donor respondent

Donors were also divided. While they were reportedly very influential, they seemed to rally behind the evidence generated from the studies that they supported, as shown in the following quote:

“*I remember some donors were in for AQ and AS, while others were in for AL. So the MoH, as the chair at that time, listened to both sides as they debated. We listened to the pros and cons of the evidence they were giving and at the end we decided to opt for AL, based on the evidence we had and other considerations. Although AQ/AS was more affordable and its efficacy was more than 90%; artemether had an efficacy of 99% and in most areas 100%. We did not want to go through another costly policy change process in the near future so we opted for AL.*” MoH respondent

The WHO reportedly had a strong influence on the MoH, as shown in the following quotes:

“*The WHO has the greatest influence on health policy in the world with relation to disease areas like HIV, malaria,* etc. *So Uganda as a country will often look at the WHO for the decisions that it makes. The WHO has some of the best doctors in the world and they are looking at all of the evidence that is published, and weighing the different evidence to try and make decisions on what makes sense. So countries rely on the decision making ability of the WHO and use that in their own decision making.*” Donor respondent

“*I remember our guidance was that any research that was conducted and funded by the WHO was most credible, because we rely on the WHO’s advice and take the WHO recommendations seriously.*” MoH respondent.

The review of documents further supported this, as the documentation of the malaria treatment policy change process in Uganda (MoH 2006) stated that “*the WHO provided technical assistance and each of the five combinations treatments recommended by WHO for the African Region at that time was considered as a possible alternative*” [42]. Although the WHO was seen as a neutral body, there were some suspicions that perhaps the WHO was being influenced by the manufacturers of AL. One donor respondent said that “the *WHO had a fair share of that suspicion because, in the interest of promoting ACTs as the most effective, the WHO went into an arrangement with the manufacturers of AL. They believed that this was one of the best ACTs, and negotiated with the manufacturer so that countries will get a reduced price. Fortunately, ACTs happened to come out as the best option for most of the countries*”. The review of documents supported this view, as the documentation of the malaria treatment policy change process in Uganda (MoH 2006) stated that “the *Project Management Unit (PMU) of GF in the MoH was mandated to procure COARTEM® (AL) through the WHO, taking into consideration the agreements between WHO and Novartis Pharmaceutical Company, which provides for subsidized COARTEM® (AL) for the public sector*”.

Communities were weak and largely non-participatory. One donor remarked that “*We don’t involve the community in policy discussions. We do not even disseminate evidence to them. We assume and choose what we think is good for them and we just take it to them*”. Indeed, among the reported roles played by the community, respondents only mentioned “beneficiaries of the policy change”.

Service providers at the national referral hospital and at the district levels were both reported as resistors, but for different reasons. At the national level, the service providers were not convinced that the change was needed. For example, one donor remarked that “*some senior consultants from Mulago hospital (national referral hospital) didn’t think that we needed to change to ACTs. They were saying that this was commercially driven*”. The service providers at the district level were apparently rather passive resistors, with one service providers stating that “*the case of non-compliant health workers was a small barrier that was eventually beaten. Some health workers resisted but eventually they had to follow*”. This may have stemmed from their lack of effective involvement in the part of the decision-making process where evidence justifying a policy change was discussed. One donor remarked that “*I can tell you that we only got views of district level service providers indirectly from the studies, but not directly. We got a few district health workers into the policy discussions but not so much beyond that*”. Service providers at the district level were not even aware of the evidence that had been discussed at the national level. One of them stated that “*we have a problem of poor dissemination of evidence. A lot of evidence is generated but is poorly presented and does not reach clinicians*”. Another service provider stated that “*there was no local evidence,* e.g. *the local laboratory network was not consulted, (…). Clinical trials must be carried out, but this was not done*”, indicating a lack of awareness of the evidence that had been discussed extensively at the national level.

Parliamentarians were weak and passive supporters, although some of them disseminated evidence to their constituents and sensitised communities about the new policy. The review of documents revealed targeted dissemination of evidence to parliamentarians. In fact, a special handbook was developed for them, which was intended to raise awareness among parliamentarians as well as to empower them with the information that they required to effectively mobilise their constituents.

### Other external influences impacting evidence uptake

Respondents raised concerns regarding the extent to which evidence can guide decision making in policy development amidst external influences. This is illustrated by the following quotes:

“*My experience is that while evidence is actually required and very strongly talked about, we also face external influences in the policy process. You actually notice that the evidence was glossed over by pressure.*” MoH respondent

“*I think there was a lot of advocacy for ACTs. If I remember well, advocacy papers were written saying that evidence has shown that there is no resistance to ACTs at all anywhere in the world. There was an advocacy letter that had been written by [Z] saying children were dying because we are treating them with CQ and not with ACTs. And then of course, there was a lot of push, given that many countries had started to use ACTs.*” Researcher respondent

In light of these external influences, respondents decried the lack of systems to manage conflicts of interest. A MoH respondent remarked that “*we don’t have systems for managing conflicts of interest. That’s the difference between the MoH and some research institutions like the Uganda National Academy of Health Sciences where I am a member. The first meeting is to prove that no one has a conflict of interest. We always have to sign and agree on the procedure.*”

## Discussion

Our present results identified a diverse group of stakeholders who played multiple roles and had varying levels of influence and support, which poses a challenge to evidence uptake. Woelk et al. documented similar challenges to evidence uptake in a case regarding efficacy of bed nets, where a diverse group of stakeholders differed in their interpretation and use of evidence, and aligned with different positions based on ideology and commercial interests [27]. The present findings also support the argument by Sumner et al. that the stakeholders’ roles and levels of influence will differ based on the nature of the policy and their interest in a given issue [43].

Our findings showed that donors, CSOs, the MoH, service providers, and researchers engaged in the role of evidence generation. Lack of evidence is a known barrier to KT [21],[39], which donors addressed by providing funding and technical assistance to undertake research. However, scholars have cautioned about remaining concerns regarding the relevance and objectivity of donor-supported research [30],[43],[44]. Indeed, in our study, we noted that the different donors tended to rally behind the evidence that was generated from the research studies that they supported.

Although the literature has highlighted that CSOs have limited capacity for evidence generation [20], we found that CSOs did participate in this area. Malik et al. also documented a successful experience where a CSO played a central role in evidence generation leading to change of the malaria treatment policy in Sudan [29]. This supports the augment by Tomlinson et al. that the roles played by the different actors will differ depending on the nature of the policy [26]. We further note that the researchers and policymakers showed close working relationships, which have previously been described as complex [21],[45]. Researchers engaged in generating evidence addressing policy-relevant research questions, and interactions with the MoH enhanced evidence uptake.

Dissemination of evidence was performed by the MoH, parliamentarians, and opinion leaders at the national and local levels. In contrast, the literature highlighted that the media, knowledge brokers, and structures either within or external to the MoH undertake such evidence dissemination [6],[9]. This difference could be explained by the present targeted dissemination of evidence and provision of advocacy materials to the parliamentarians and local leaders. The involvement of local leaders could also explain how the communities became aware of the policy change [46] despite their lack of systematic involvement in evidence generation and decision making. The lack of community participation in KT is a long-standing concern and occurs partly due to the absence of appropriate structures for meaningful community engagement [44],[46]. In this regard, opinion leaders are potential stakeholders who can support evidence dissemination and subsequently improve KT. The present study also suggested a need to further explore the role of parliamentarians in KT.

The role of evidence uptake in policy development was played by donors, the MoH, researchers, and service providers through representation. Uptake of evidence performed by donors has been described as supportive or disruptive, depending on the policy in question, their interests, and the nature of the evidence [47]. One relevant concern is that significant financial flows to support health programmes may give the donors undue influence in decision making [48]. Cases have been documented in which policy decisions were made based on the donors’ influence despite available evidence supporting alternative decisions [29],[46],[49]. This was also noted in our study, as one respondent stated that Global Fund “*…were saying the Global Fund grants should only be used for buying ACTs…*”, while the MoH was relying on the Global Fund round 4 to fund implementation of the new policy. Challenges of donor dependency have also been reported in other countries. For instance, Cameroon’s national efforts to coordinate research were undermined [50] and, in Ghana, changes in HIV treatment guidelines were influenced by the conditions of donor financing [49]. Hutchinson et al. similarly reported that the donors’ active involvement in evidence generation and policy development influenced decision making [51].

Some have argued that the MoH should lead the KT process in order to ensure focus on country priorities [52],[53]. In our present study, the top management and technical programmes within the MoH led the policy development process and considered evidence in decision making. However, it was of concern that MoH officials were divided regarding the decision to change and what alternative first-line antimalarial to adopt. This may be explained by several reasons. Firstly, although some MoH officials followed the research process, they may not have had a specific position on which evidence to adopt, as it was reported that “…*the chair at that time listened to both sides as they debated. We listened to the pros and cons of the evidence they were giving…*”. Secondly, evidence may not have been comprehensive enough to allow decision making, which was supported by the statement that “*…and at the end we decided to opt for AL, based on the evidence we had and other considerations*”. Similarly, Mubyazi and Gonzalez-Block [16] documented instances where the decision to change the malaria treatment policy was protracted as decision makers kept requesting more evidence on different aspects of policy development. The policy process analysed in the present study took 25 months.

The top management cadre of the MoH that was ultimately responsible for decision making was noted to be a weak and passive supporter, which could be explained by their limited involvement in the research processes. Panisset et al. documented successful experiences in Bangladesh, in which the involvement of the Directorate General of Health Services in the research process enhanced the uptake of zinc for use in diarrhoea management among young children [28]. Another possible explanation could be that the top management officials had different views regarding what the best alternative was, partly influenced by the different positions of the technical programmes. This implies that realisation of successful KT requires that the technical teams that guide top management must be convinced about the evidence and must reach a consensus on possible policy options. Additionally, despite time constraints, the top management have to take a keen interest in evidence synthesis and interpretation. The time constraints could be alleviated by provision of evidence in brief digestible formats and the use of advisors and think tanks [47],[54].

The participation of researchers in the policy development process improved evidence uptake [14]. Although the researchers were divided regarding the best alternative to adopt, their participation in the policy formulation and decision making, including discussions of the different options, enabled consideration of other evidence relevant to this decision. Indeed, health expenditure trends were discussed alongside efficacy data to assess affordability.

In the majority of countries, implementation of evidence is performed by the MoH, public and private service providers, and CSOs [20]. However, in our present case study, the role of private for profit providers appears to have been maximised, as similarly noted in other studies of KT in Uganda [55],[56].

It has been reported that actors play different roles depending on the nature of the policy and their level of interest in a given issue [26],[27],[43], and our present findings support this position. For example, in our earlier study of actors in KT in Uganda [30], CSOs were not reported to engage in evidence generation. This may be explained by the varied technical capacity of CSOs, in that they may internally possess the skills required to engage in certain types of research processes, but not in all cases. Indeed, Pollard et al. have noted that the varied capacity of CSOs is a limitation to their effective engagement [20]. Our earlier study of the roles of actors in KT also reported that CSOs and the media engaged in the dissemination of evidence. Their failure to engage in dissemination of evidence in the malaria treatment change may have been due to the absence of targeted dissemination. The literature emphasises the importance of providing information in a simple and clear format [12], which was not done in our present case study. On the other hand, donors and researchers were not reported as actors in the policy process, yet we noted their participation in the case of malaria treatment policy change.

In our present study, some respondents decried the lack of systems to manage conflicts of interest. Boyd and Bero emphasised the importance of identifying and managing conflict of interest in order to improve evidence uptake [57]. The proposed steps include soliciting information from actors regarding possible conflict of interest, having explicit criteria to determine whether the disclosed financial or other competing interest indeed constitute a conflict of interest, and finally, providing guidance on how confirmed conflicts should be managed.

### Policy implications

Stakeholders in KT play different roles and exert different levels of influence and support based on the nature of the policy and the evidence under consideration. This raises the need to map relevant stakeholders and to work out mechanisms for their involvement and for how evidence can be utilised by all in an objective manner. Provision of regular updates along research processes and targeted dissemination of evidence beyond in-country stakeholders may enhance inclusiveness and consensus building. Additionally, given the multiple array of stakeholders, there is a need to utilise mechanisms to manage conflict of interest.

### Strengths and weaknesses of the study

Our study has several strengths. Firstly, we interviewed a wide array of stakeholders and used multiple data collection methods, which provided a rich data set to use in assessing the roles and influence of stakeholders in evidence uptake. Secondly, the multidisciplinary nature of the research team helped to ensure objectivity in the data analysis and interpretation. Thirdly, interviews were conducted by the first author who is experienced with policy development in Uganda.

The study also had several weaknesses. Firstly, recall bias may have impacted the accuracy of the responses, given the timing of the policy process and data collection. However, we noted consistency among the responses, suggesting that perhaps recall bias was not as much of a problem as might be anticipated. Secondly, with the exception of researchers, we considered stakeholders to be organisations/institutions/units. It should be noted that individuals may not necessarily represent the views of the institution in which they work; however, this was not explored in our study. Thirdly, it is possible that the stakeholders’ levels of influence and support in the uptake of evidence in policy could change at the different stages of policy development, but this was also not assessed in our study.

## Conclusions

Stakeholders played multiple roles in the uptake of evidence in the malaria treatment policy change in Uganda. The donors, MoH, service providers, and researchers engaged in the role of evidence generation. The MoH, parliamentarians, and opinion leaders at the national and local levels engaged in dissemination of evidence. The donors, MoH, researchers, and service providers through representation engaged in the uptake of evidence in policy development and implementation. Stakeholders exerted varying levels of support and influence for different reasons. For example, donors were influential due to their significant financial support, researchers were influential because they commanded respect, and the MoH was influential because the uptake of evidence and subsequent policy implementation heavily depended on them. It is noteworthy that all of the influential actors were divided regarding the best alternative antimalarial to adopt.

Our findings suggest that the roles and influence of stakeholders in KT will vary given the nature of the policy and the available evidence. For a given KT process, mapping the relevant stakeholders and devising mechanism for their engagement and for resolving conflicts of interest will enhance the uptake of evidence in policy development. Additionally, structures and systems must be put in place to encourage community participation in research processes and decision making.

## Additional files

## Electronic supplementary material

Additional file 1: Documents reviewed: policies, guidelines, meeting minutes, position papers, research report. Details of milestones and list of documents reviewed. (DOC 52 KB)

Additional file 2: Examples of how manifest content and thematic content analyses were performed. (a) Example of how manifest content analysis was performed. Details of how data was analysed to come with categories. (b) Example of how thematic content analysis was performed. Details of how data was analysed to develop themes. (ZIP 16 KB)

Below are the links to the authors’ original submitted files for images.

Authors’ original file for figure 1

Authors’ original file for figure 2

## Abbreviations

ACTs: artemisinin combination therapy
AL: artemether-lumefantrine
CQ: chloroquine
CSOs: civil society organisations
GF: Global Fund
JMS: Joint Medical Stores
KT: knowledge translation
MoF: Ministry of Finance
MoH: Ministry of Health
NDA: National Drug Authority
NMS: National Medical Stores
SP: sulfadoxine/pyrimethamine
