# Supplementary figures and images for: The roles and influence of actors in the uptake of evidence: the case of malaria treatment policy change in Uganda
Source: Implement Sci. 2014 Oct 8;9:150. doi: 10.1186/s13012-014-0150-8 (PMC4193992; doi:10.1186/s13012-014-0150-8)

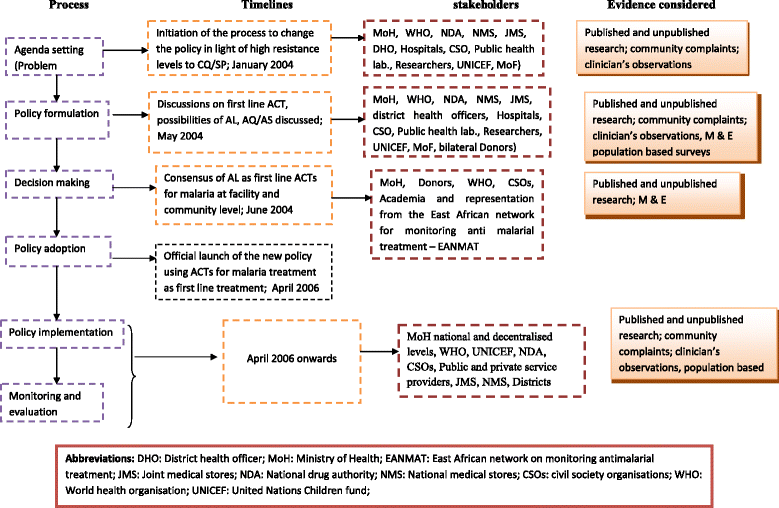

Supplement: Supplementary file 3 — Authors’ original file for figure 1 [file 13012_2014_150_MOESM3_ESM.gif]

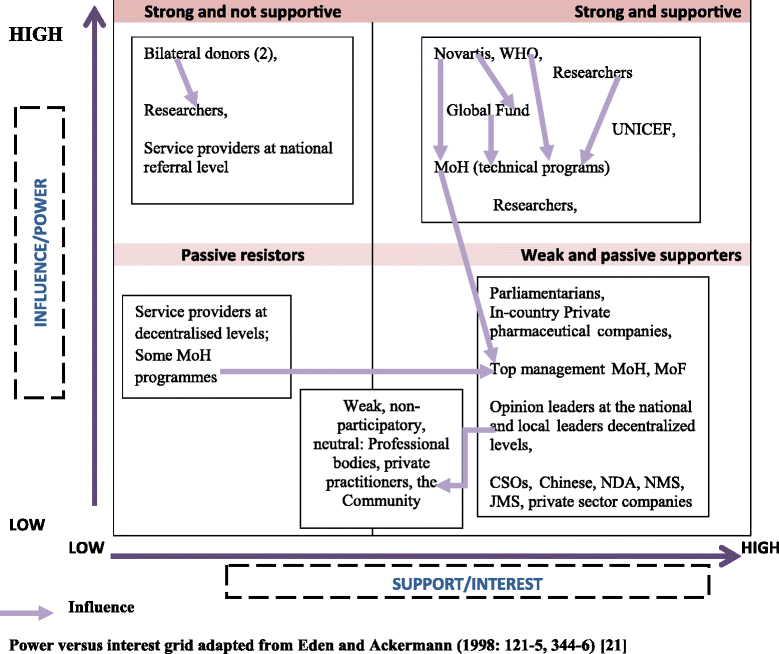

Supplement: Supplementary file 4 — Authors’ original file for figure 2 [file 13012_2014_150_MOESM4_ESM.gif]
