# Supplementary material for: The roles and influence of actors in the uptake of evidence: the case of malaria treatment policy change in Uganda
Source: Implement Sci. 2014 Oct 8;9:150. doi: 10.1186/s13012-014-0150-8 (PMC4193992; doi:10.1186/s13012-014-0150-8)
Supplement: Supplementary file 2 — Additional file 2: Examples of how manifest content and thematic content analyses were performed. (a) Example of how manifest content analysis was performed. Details of how data was analysed to come with categories. (b) Example of how thematic content analysis was performed. Details of how data was analysed to develop themes. (ZIP 16 KB) [file 13012_2014_150_MOESM2_ESM.zip › add2/7477237671241477_add2a.doc]

# Additional file 2a Example of how manifest content analysis was performed

| **Roles of stakeholders** | | **Condensed meaning** | **Category** |
| --- | --- | --- | --- |
| WHO | The WHO has the capacity to generate evidence on malaria drug resistance, they have been doing this through the years. They advise ministries of health and advised the MoH on the most appropriate decision, although some people rated the evidence differently. | The WHO has capacity to generate required evidence | Providing evidence |
|  | The WHO also provided support to sentinel sites which were undertaking efficacy studies, this actually increased the credibility of the research results because the methodologies were standardised as these were multi-country studies. And WHO is respected in this kind of research on efficacy of medicines. | Provided technical support for undertaking research |  |
| Technical programmes in the MoH | The National Malaria Control Programme are all under the ministry of health. They advise on malaria management and are actually the ones that drafted the policy | Designing the policy | Policy development |
|  | The key stakeholder was the ministry of health, especially for the malaria control program. They were the ones who were going to develop and implement the policy | Responsible for developing and implementing the policy |  |
|  | The reproductive health programme and the pharmaceutical division participated actively in the policy development process. They actually chaired some of the working groups. | Participated in policy development |  |
| CSOs | Some civil society organisations were part of the working group that deliberated on the policy and also reviewed the different drafts of the policy. | Deliberated on the policy and reviewed drafts | Participating in policy discussions |
|  | CSOs participated in the policy process. | Participated in the policy process |  |
