# Supplementary material for: The roles and influence of actors in the uptake of evidence: the case of malaria treatment policy change in Uganda
Source: Implement Sci. 2014 Oct 8;9:150. doi: 10.1186/s13012-014-0150-8 (PMC4193992; doi:10.1186/s13012-014-0150-8)
Supplement: Supplementary file 2 — Additional file 2: Examples of how manifest content and thematic content analyses were performed. (a) Example of how manifest content analysis was performed. Details of how data was analysed to come with categories. (b) Example of how thematic content analysis was performed. Details of how data was analysed to develop themes. (ZIP 16 KB) [file 13012_2014_150_MOESM2_ESM.zip › add2/7477237671241477_add2b.doc]

**Additional file 2b Example of how thematic content analysis was performed**

| **Level of influence** | |  |  |  |
| --- | --- | --- | --- | --- |
|  |  | **Condensed meaning** | **Category** | **Theme** |
| WHO | The WHO has the greatest influence on policy, and is viewed as the premier policy-making body in the world with regard to disease areas like HIV, malaria, etc. Thus, Uganda as a country will often look towards the WHO for the decisions that it makes. They also advise other ministries of health for example on TB and other communicable and non communicable disease. | Has the greatest influence on policy | Strong influence on policy | Strong and influential |
|  | WHO is very influential on health matters because it is a health specialised agency among UN agencies.Countries rely on the decision-making ability of the WHO and use evidence and guidance provided by WHO in their own decision making. | Countries rely on the decision making ability of the WHO | Reliance on WHO |  |
|  | The WHO has some of the best doctors in the world, who are all examining all of the published evidence, and weighing these different pieces of evidence to try and make reasonable decisions. | WHO has the best technical capacity | WHO has technical capacity |  |
|  | International bodies. Basically, the ministry of health is influenced by evidence and guidance provided by WHO. | Ministry of health is influenced by WHO | WHO influences the MoH |  |
|  | The WHO, because our interest was always to make sure that whatever we came up with for the change in the existing policy was not going to contradict with them. The WHO were strongly supportive. | Aligning with WHO advice | MoH reliance on WHO |  |
| Communities | Largely we don’t involve the community in policy research processes and policy discussions, and we assume that we can determine what is good for them. Thus, we choose what is good for them, and we talk for them. They were receptive because the research showed that this was a good drug, and they were happy, but they did not participate at all in the processes. | Communities were not involved and did not participate in the policy process | Communities did not participate in the policy process | Weak and non-participatory |
|  | The community did not participate in the policy process because we do not have systems in place to help them participate, and this actually weakens the community. Even in research processes, they are respondents when researchers go to them | Lack of systems to enable community participation weakens communities | Lack of systems for community participation |  |
|  | Our communities are weak and we do not have a very good public opinion, and I don’t know whether we have a system in which we can interview communities about these drug changes. I don’t think I have seen much of that. | Lack of consultative mechanisms to gather community views on medicines | Weak communities with no systems in place for engagement |  |
